# The convergence of radiation and immunogenic cell death signaling pathways

**DOI:** 10.3389/fonc.2012.00088

**Published:** 2012-08-07

**Authors:** Encouse B. Golden, Ilenia Pellicciotta, Sandra Demaria, Mary H. Barcellos-Hoff, Silvia C. Formenti

**Affiliations:** ^1^Department of Radiation Oncology, New York UniversityNew York, NY, USA; ^2^Department of Pathology, New York UniversityNew York, NY, USA

**Keywords:** ionizing radiation, immunogenic cell death, apoptosis, necrosis, autophagy, mitotic catastrophe, senescence

## Abstract

Ionizing radiation (IR) triggers programmed cell death in tumor cells through a variety of highly regulated processes. Radiation-induced tumor cell death has been studied extensively *in vitro* and is widely attributed to multiple distinct mechanisms, including apoptosis, necrosis, mitotic catastrophe (MC), autophagy, and senescence, which may occur concurrently. When considering tumor cell death in the context of an organism, an emerging body of evidence suggests there is a reciprocal relationship in which radiation stimulates the immune system, which in turn contributes to tumor cell kill. As a result, traditional measurements of radiation-induced tumor cell death, *in vitro*, fail to represent the extent of clinically observed responses, including reductions in loco-regional failure rates and improvements in metastases free and overall survival. Hence, understanding the immunological responses to the type of radiation-induced cell death is critical. In this review, the mechanisms of radiation-induced tumor cell death are described, with particular focus on immunogenic cell death (ICD). Strategies combining radiotherapy with specific chemotherapies or immunotherapies capable of inducing a repertoire of cancer specific immunogens might potentiate tumor control not only by enhancing cell kill but also through the induction of a successful anti-tumor vaccination that improves patient survival.

## Introduction

Radiation therapy (RT) is a well-established and effective form of cancer treatment. Since radiotherapy effects within an irradiated field are not tumor specific, the therapeutic ratio depends on the ability to localize ionizing radiation (IR) delivery to the tumor site and optimize dose and fractionation to preferentially kill tumor cells better than exposed normal cells.

IR can directly damage the atomic structures of nucleic acids, proteins, and lipids. In addition, molecular damage is indirectly mediated by byproducts of radiation exposure, consisting of free radicals produced from water radiolysis. Both the direct and indirect effects of IR initiate a series of downstream signaling events that result in either the repair of damaged macromolecules or evolve toward some form of cell death. The detrimental effects of radiation depend on both the dose and efficiency of damage repair of the irradiated target. In tumor cells, the most biologically sensitive and clinically relevant macromolecule influencing cell death is DNA, which is susceptible to single-strand breaks (SSBs) and double-strand breaks (DSBs) (Giusti et al., 1998). It is estimated that 1 Gy can produce 20–40 DSBs per cell, where unrepaired DSBs can result in cellular lethality (Jonathan et al., 1999; Schultz et al., 2000).

After radiation exposure, tumor cells undergo different types of tumor cell death, including: apoptosis, necrosis, mitotic catastrophe (MC), autophagy, and senescence (Gudkov and Komarova, 2003; Eriksson and Stigbrand, 2010). The type of cell death depends on several interrelated factors. These factors include the cell type, radiation dose and quality, oxygen tension, p53 mutation status, DNA repair capacity, redox state, and the cell cycle phase at the time of IR exposure (Stewart et al., 2011). In addition, different types of cell death pathways interact to contribute to the final outcome within the irradiated tumor.

The classical pathways of IR-induced cell death well described *in vitro* fail to adequately explain all *in vivo* experimental and clinical observations. An abscopal (*ab scopus*, away from the target) response is perhaps the most convincing evidence that direct DNA damage is not the only mechanism of tumor control. This is supported clinically when radiation is delivered focally and tumor response is systemic, for example when metastatic tumors outside of the treated field respond to treatment (Nobler, 1969; Ehlers and Fridman, 1973; Ohba et al., 1998; Takaya et al., 2007).

Evidence in experimental models suggests that radiation-induced promotion of anti-tumor immune responses can explain these abscopal effects (Chakravarty et al., 1999; Demaria et al., 2004; Shiraishi et al., 2008; Dewan et al., 2009). However, a gap exists in bridging the current understanding of principles in radiation biology and this effect of IR on immune activation. Immunogenic cell death (ICD) has become a topic of discussion to both explain initiating events and optimize the clinical benefit of the abscopal effect. This review will focus on the modes of tumor cell death following IR, the methods used to interrogate cell death modalities, and the consequences of cell death on tumor-host interactions. Additional effects of RT on the tumor microenvironment have been reviewed elsewhere (Gudkov and Komarova, 2003; Barcellos-Hoff et al., 2005).

## Apoptosis

Apoptosis is a highly regulated mechanism of programmed cell death that plays a fundamental role in embryonic development and tissue homeostasis to eliminate unwanted, damaged, or abnormal cells. Cells undergoing apoptosis are characterized by distinct cytoplasmic and nuclear morphologic changes, including membrane blebbing, DNA fragmentation and nuclear condensation. Dysregulation of apoptosis, however, is associated with unchecked cell proliferation and is thought to be essential for the development and progression of cancer (Fuchs and Steller, 2011). Thus, therapies that augment apoptosis have become a powerful tool in treating cancer.

The apoptotic pathway comprises a complex network of proteins that are cell type and spatiotemporally dependent. Genes involved in apoptosis may act in concert or show redundancy (Kuribayashi et al., 2011). Nonetheless, distinct apoptotic pathways have been clearly defined in IR exposed tumor cells.

**Figure 1:**
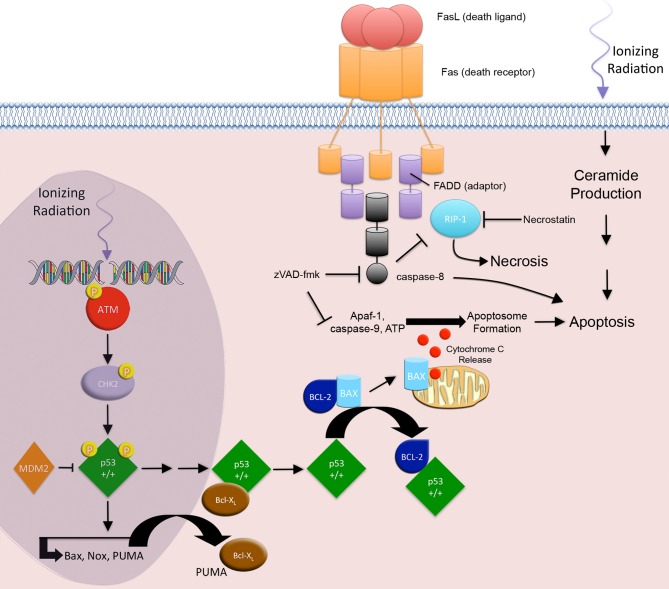
**Apoptosis and necrosis.** IR-induced apoptosis through the intrinsic apoptotic pathway begins with the development of DNA DSBs. ATM and CHK2 associated p53 phosphorylation events result in the nuclear accumulation of transcriptionally active p53, which in turn transactivates the pro-apoptotic genes PUMA, Bax, and Noxa. Cytoplasmic PUMA disrupts the Bcl-X_L_/p53 complex and liberates p53. Free cytoplasmic p53 disrupts the Bcl2/Bax complex by associating with the anti-apoptotic protein Bcl2 and releasing the pro-apoptotic protein Bax. Bax triggers cell death through the release of cytochrome c from the mitochondria, resulting in apoptosome formation and activation effector caspases and apoptosis. The extrinsic pathway involves signaling through death receptors. IR activation of p53, results in downstream transactivation of CD95/Fas and the CD95/Fas ligand. CD95/Fas ligand binding to CD95/Fas induces trimerization and clustering of the intracellular death domain (DD) region of the receptor. The DD recruits the adaptor protein, Fas-associated death domain (FADD). The death effector domain of FADD recruits pro-caspase-8, forming the death-inducing signaling complex. Activation of the initiator caspase-8 leads to activation of effector caspases and apoptosis. Additionally, IR-induced membrane stress leads to ceramide production, second messenger signaling, and apoptosis. RIP1 protein is associated with the FADD and is a key upstream kinase involved in the activation of regulated necrosis. Regulated necrosis is sustained in death receptor stimulated cells that are caspase-8 deficient or inhibited.

Depending on dose and cell type, RT may cause apoptosis via the membrane stress pathway (ceramide production and subsequent second messenger signaling), the intrinsic pathway (mitochondrial release of cytochrome and subsequent apoptosome formation), and the extrinsic pathway (death receptor mediated caspase activation) (Figure 1) (Cain et al., 1999; Ogura et al., 2009). IR primarily acts through the intrinsic pathway, but it has also been shown to involve certain aspects of the membrane stress and extrinsic pathways (Takasawa et al., 2005).

IR-induced apoptosis through the intrinsic apoptotic pathway is mediated by DNA SSBs and DSBs (Gudkov and Komarova, 2003). This damage elicits subsequent downstream signaling to either block cell cycle progression, allowing for DNA repair, or progression to cell death when DNA damage is overwhelming. ATM and ATR activation mediate the early responses to IR that regulate cell cycle progression and DNA repair (Maltzman and Czyzyk, 1984). In the presence of DSBs, Mre11/Rad50/Nbs1 complexes form at DSB sites and recruit ATM to sites of repair. ATM undergoes autophosphorylation and phosphorylates checkpoint protein kinase 2 (Chk2) (Smith et al., 2010; Rodriguez-Rocha et al., 2011). At SSB sites, Rad1/Rad9/Hus1 and Rad17/RFC complexes form and recruit ATR. ATR undergoes autophosphorylation, and soon after phosphorylates checkpoint protein kinase 1 (Chk1) (Smith et al., 2010; Rodriguez-Rocha et al., 2011). Activated Chk1 and Chk2 block tumor cell cycle progression by regulating DNA repair and cell cycle proteins, including BRCA1, MDM2, and p53 (Cortez et al., 1999; Kim et al., 2002; Lukas et al., 2004; Shi et al., 2004).

The accumulation of p53 is critical to IR-induced apoptosis. Activated ATM phosphorylates nuclear p53 protein on serine 15, thereby preventing its ubiquitination by MDM2 and subsequent proteasomal degradation (Siliciano et al., 1997; Dumaz and Meek, 1999; Tichy et al., 2009). Additionally, ATM/ATR-activated Chk1 and Chk2 kinases phosphorylate the p53 transactivation domain on serine 20, thereby stimulating p53 activity (Dornan et al., 2003). These p53 phosphorylation events result in the nuclear accumulation of transcriptionally active p53, which in turn transactivates the pro-apoptotic genes PUMA, Bax, and Noxa (Oda et al., 2000; Dogu and Diaz, 2009; Kuribayashi et al., 2011).

A fine balance regulates this pathway. In the cytoplasm, p53 is associated with the anti-apoptotic protein Bcl-X_L_. When PUMA is translocated into the cytoplasm it disrupts the Bcl-X_L_/p53 complex and liberates p53 (Chipuk et al., 2005). Free cytoplasmic p53 disrupts the Bcl2/Bax complex by associating with the anti-apoptotic protein Bcl2 and releasing the pro-apoptotic protein Bax.

Bax induces permeabilization of the outer mitochondrial membrane to trigger cell death through the release of cytochrome c from the mitochondria (Marzo et al., 1998; Dejean et al., 2006; Dogu and Diaz, 2009). In the cytoplasm, cytochrome c, Apaf-1, and ATP form the apoptosome and activate caspase-9, thereby initiating the postmitochondrial-mediated caspase cascade by activating effector caspases 3 and 7 (Cain et al., 1999).

In addition to the intrinsic apoptotic pathway, IR is also involved in the canonical extrinsic apoptotic pathway. The classic apoptotic machinery of the extrinsic pathway involves signaling through death receptors (DRs), which belong to the tumor necrosis factor (TNF) receptor superfamily.

IR activation of p53, results in downstream transactivation of CD95/Fas, KILLER/DR5, and the CD95/Fas ligand (CD178) (Sheard, 2001; Harms et al., 2004). CD95/Fas ligand binding to CD95/Fas induces trimerization and clustering of the intracellular death domain (DD) region of the receptor. The DD recruits the adaptor protein, Fas-associated death domain (FADD) (Sheard, 2001). The death effector domain (DED) of FADD recruits pro-caspase-8, forming the death-inducing signaling complex (DISC). Activation of the initiator caspase-8 leads to activation of effector caspases 3 and 7, which act to disassemble cellular structures. Interestingly, IR associated up-regulation of CD95/Fas on tumor cells improves tumor cell kill by effector CD8+ cytotoxic T lymphocytes (CTLs) that express CD95/Fas ligand and may also play a role in delayed apoptosis associated MC (Luce et al., 2009).

Strategies aimed at augmenting apoptosis constitute a common research area in oncology. However, p53 is mutated in ~50% of cancers and the apoptotic machinery is defective in most others, influencing responses to IR. In fact, tumors that are susceptible to p53 dependent apoptosis are quite radiosensitive, whereas, tumors that overexpress antiapoptotic proteins (BCL2, Bcl-X_L_, and Survivin) or lose expression of proteins involved in the apoptotic machinery are radioresistant (Cuddihy and Bristow, 2004; Rodel et al., 2005).

In cancers with p53 mutations, unchecked cell proliferation occurs in spite of DNA damage by IR. When this happens, the tumor cells accumulate DNA mutations, become aneuploid, and develop micronuclei, leading to MC and subsequent cell death (Lane, 1992). Interestingly, MC is frequently followed by delayed apoptosis in apoptosis-competent cells (Gudkov and Komarova, 2003). Since p53 mutation is frequently seen in tumor cells, MC may be the predominant form of cell death following IR exposure to tumor cells, even though this effect is cell-type dependent.

## Necrosis

Necrosis is a tumor cell death pathway that predominates in response to very large doses of radiation. At lower doses it is often viewed as an accidental, unregulated event. In contrast to apoptosis, necrosis does not display signs of ordered DNA fragmentation. Necrotic morphology is evident in studies using light or electron microscopy. Cells display the morphological features of organelle swelling, mitochondrial dysfunction, and plasma membrane permeabilization with subsequent loss of intracellular contents, including immune stimulating “danger signals” (Galluzzi and Kroemer, 2008; Hotchkiss et al., 2009).

IR-induced necrosis in tumor cells is not to be confused with the indirect effects of IR occurring as untoward toxic effects in the clinic, like delayed osteoradionecrosis or central nervous system (CNS) radiation necrosis after high dose IR exposure of normal bone or brain tissue, respectively (Chrcanovic et al., 2010; Fink et al., 2012; Siu et al., 2012). These indirect effects of IR are mediated by vascular dysfunction, thereby making normal cells susceptible to undergoing necrosis due to hypoxia and nutrient depletion (Garcia-Barros et al., 2003; Teng and Futran, 2005; Ruegg et al., 2011).

**Figure 2:**
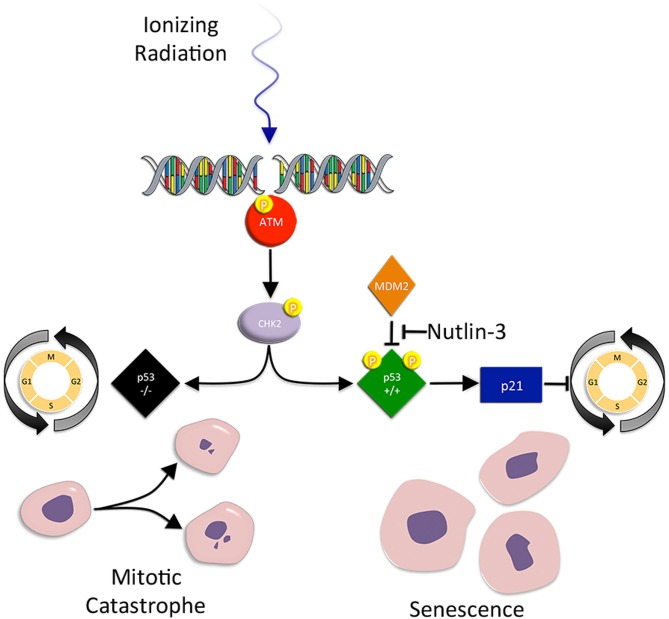
**Mitotic catastrophe and senescence.** Mitotic catastrophe (MC) occurs after failed mitosis. Cells are characterized by an increased frequency of multiple nuclei and micronuclei. Checkpoint control is greatly reduced in mutant p53 tumor cells, making the cells susceptible to premature mitosis, chromosomal dysegregation, the generation of aneuploid progeny, and MC associated cell death. Senescence on the other hand, is a form of irreversible growth arrest that halts the proliferation of metabolically active cells. IR-induced accelerated senescence in tumor cells is centered around p53. When IR induced DSBs result in p53 activation, activated p53 promotes the activity of p21, an inhibitor of CDK/cyclin complexes and cell cycle progression. Senescent cells display the loss of replicative potential, cell cycle arrest, and enlarged and flattened morphology.

Recent literature suggests that IR can directly induce regulated tumor cell necrosis (Nehs et al., 2011). Programmed necrosis (necroptosis) displays some overlap with apoptosis. It is a cellular mechanism of necrotic cell death induced by apoptotic stimuli, i.e., ligand-DR engagement, under conditions where the apoptotic machinery is either deficient or blocked (Figure 2) (Degterev et al., 2008). Both apoptosis and necrosis share components of the DR signaling apparatus, specifically at the level of the FADD (Stanger et al., 1995; Vanden Berghe et al., 2004). In both forms of cell death, the deciding factor of whether a cell commits apoptosis or necrosis depends on the FADD associated activities of caspase-8 and receptor interacting protein 1 (RIP1) (Lin et al., 1999; Holler et al., 2000).

RIP1 is a key upstream kinase involved in the activation of necroptosis (Degterev et al., 2008). It interacts with the DD of FADD and is regulated by its ubiquitination and cleavage states (Vanden Berghe et al., 2004; Declercq et al., 2009). When RIP1 is polyubiquitinated it functions as a pro-survival scaffold and promotes downstream activation of mitogen-activated protein kinases (MAPKs) and NFκ-B, which both govern the expression of pro-survival genes (Declercq et al., 2009). However, upon RIP1 polyubiquitin chain removal, RIP1 associated MAPK and NFκ-B activation is abolished, and RIP1 downstream necroptotic signaling is preferentially promoted through the mitochondrial permeability transition complex, as opposed to mitochondrial outer membrane permeabilization transition complex, seen in apoptosis (Declercq et al., 2009). This effect is sustained in DR stimulated cells that are caspase-8 deficient or inhibited (blocked by pancaspase inhibitors, i.e., zVAD-fmk). Nevertheless, if caspase-8 is intact and active, it can cleave RIP1, thereby turning off necroptosis, and alter the balance of cell death in favor of apoptosis (Lin et al., 1999).

Recent work by Nehs et al. demonstrated that necroptosis contributed to IR-induced cell death of anaplastic thyroid and adrenocortical cancers (Nehs et al., 2011). They showed that IR-induced cell death could be abrogated with necrostatin-1, a small molecular inhibitor of RIP1, in RIP1 expressing tumor cells (Degterev et al., 2008; Nehs et al., 2011). They proposed that necroptosis augmentation, involving an activator of RIP1 kinase or its downstream effectors, might radiosensitize cells. However, further studies are required to clarify the role of necroptosis in IR-induced cell death and the subsequent spillage of immune stimulating “danger signals” (Nehs et al., 2011).

## Mitotic catastrophe

MC occurs after failed mitosis. Cells are characterized by an increased frequency of multiple nuclei and micronuclei. MC acts as an oncosuppresive mechanism for the avoidance of genomic instability (Vitale et al., 2011). Tumor cells that undergo MC often have checkpoint deficiencies that result in incomplete DNA repair, replicative infidelity, and chromosomal dysegregation (Eriksson and Stigbrand, 2010). Thus, loss of checkpoint control in IR exposed tumor cells eventually leads to the generation of aneuploid progeny and MC associated cell death (Ianzini et al., 2006).

Mutant p53 tumor cells are susceptible to IR-induced MC (Ianzini et al., 2006; Eriksson and Stigbrand, 2010). Normally, p53 acts as a post-transcriptional negative regulator of cyclin B1 protein (a cell cycle regulated protein that abrogates the G2/M checkpoint) levels and centrosome amplification. However, in p53 mutant cells, cyclin B1 levels are elevated and centrosome frequency is amplified (Eriksson and Stigbrand, 2010). This contributes to both premature mitosis and chromosomal dysegregation, leading to MC. Not surprisingly, inhibition of other G2 checkpoint proteins (ATM, ATR, Chk1, Chk2, and p21) promotes DNA damage, aneuploidy, and MC (Castedo et al., 2004; Hirose et al., 2005; Vogel et al., 2007).

Interestingly, MC is associated with delayed apoptosis in irradiated tumor cells. An increase in CD95/Fas, TRAIL-R, and TNF-R expression and sensitization to early apoptosis heralds a delayed increase in FasL, TRAIL, and TNFα expression and results in the execution of delayed apoptosis linked to MC (Luce et al., 2009). Not only are the ligands expressed on the surface of tumor cells, but they are also produced in the soluble form, resulting in death of ligand sensitive bystander tumor cells (Luce et al., 2009).

Recently, caspase-2 has been identified as an initiator caspase following DNA damage and is activated during apoptosis following MC (Vitale et al., 2011). However, some researchers believe that caspase-2, at best, is an amplifier of the apoptotic cascade and may not be relevant to apoptosis at all (Krumschnabel et al., 2009). Moreover, some evidence suggests that that MC may promote necrosis (rather than apoptosis) (Vakifahmetoglu et al., 2008). Since the concept of regulated necrosis is gaining consensus, attempts at understanding its relationship to IR-induced MC may prove important.

## Senescence

Senescence is a form of irreversible growth arrest that halts the proliferation of metabolically active ageing and damaged cells. Similar to other forms of cell death, it is a process that prevents the transmission of damaged genetic material to daughter cells. Several key features distinguish senescent cells, including the loss of replicative potential, cell cycle arrest, enlarged and flattened morphology, and expression of senescence-associated markers (for example senescence-associated β-galactosidase, SA-β-gal) (Suzuki et al., 2001). IR has been reported to promote accelerated senescence in normal and cancer cells (Mendonca et al., 2011). Indeed, the progeny of irradiated cells accumulate structural chromosomal aberrations in a dose dependent fashion, which precedes senescence (Zahnreich et al., 2010). Similar to other forms of cell death, p53 plays a central role in IR-induced accelerated senescence in tumor cells (Jones et al., 2005; Quick and Gewirtz, 2006; Lehmann et al., 2007).

Senescence is an option exercised in normal epithelial cells during aging, where the process is well described. In brief, p53 activation promotes activity of p21, which acts to block CDK/cyclin complexes and cause G1 cell cycle arrest. This effect is paralleled by p53 suppression of cyclin B1 expression during IR-induced G2 cell cycle arrest. Subsequent to p21 induction, p16 expression is induced, while p21 levels decline. It is recognized that p21 is involved in the initial induction of G1 arrest and p16 is required for its extended maintenance, whereby p16 prevents CDK4 and CDK6 from phosphorylating Rb protein, which binds E2F and prevents transcription of genes required for cell cycle progression. As expected, inactivation of DNA damage checkpoint kinases prevents senescence and restores cell cycle progression (Fagagna et al., 2003).

The telomere also plays a crucial role in IR-induced senescence of cancer cells (Crompton, 1997). Telomeres consist of short, highly repetitive DNA sequences located at the ends of chromosomes. Telomere length is maintained by telomerase (a complex consisting of a reverse transcriptase and RNA template). In tumor cells, IR produces chromosome end associated abnormalities, including end-to-end fusions (an indicator of telomere dysfunction) (Jones et al., 2005). Telomere dysfunction, rather than changes in telomerase activity or telomere length, induces senescence in a p53 dependent manner (Jones et al., 2005). In contrast, p53 mutant cells are unable to arrest and succumb to other forms of cell death, including apoptosis, necrosis, autophagy, and MC (Jones et al., 2005; Lehmann et al., 2007). Interestingly, nutlin-3, a small molecular p53 activator, was shown to be an effective radiosensitizer, and its effect was entirely attributable to an increased induction of p53 dependent cellular senescence in prostate cancer cells (Lehmann et al., 2007).

Senescent cells have a distinct secretory repertoire called senescence associated secretory phenotype or SASP (Coppe et al., 2010). Recent studies in liver cancer and sarcoma mouse models suggest that reactivation of p53 in p53-deficient tumors *in vivo* produces complete tumor regression predominately due to senescence induction (Ventura et al., 2007; Xue et al., 2007). These studies demonstrate that cellular senescence can limit tumor growth and may contribute to improved long-term survival. In fact, SASP mediated inflammatory cytokines may activate the innate immune system as a mediator of tumor regression (Xue et al., 2007). Again, the relationship between IR-induced senescence and an immune host response to tumor cells has not been established.

## Autophagy

Autophagy is characterized by the segregation of damaged or unwanted ER and cytoplasmic constituents into autophagosomes, destined for lysosomal degradation. It is paradoxical as it is actually a survival mechanism that induces a particular type of death when overstimulated. Autophagy is noted for its role in maintaining metabolic homeostasis in tumor cells undergoing chronic hypoxia and nutrient depletion (Bursch et al., 2008; Munz, 2009; Orvedahl and Levine, 2009). Yet, its effects are two-fold (Tsuchihara et al., 2009; Palumbo and Comincini, 2012; Wu et al., 2012). Low to moderate levels of autophagy enhance cell growth and repair by altering the cellular composition and generating building blocks available for the biosynthesis of complex molecules. Next to the proteasome, autophagy is an important catabolic pathway necessary for recycling amino acid, fatty acid, and energy (in the form of ATP) (Munz, 2009; Rodriguez-Rocha et al., 2011). In contrast, hyper-activation of autophagy promotes cell death, when degradation of cytoplasmic contents proceeds to completion (Huang and Klionsky, 2007; Chen and Karantza-Wadsworth, 2009).

While IR has been shown to induce autophagy in tumor cells, the literature is conflicting, regarding whether IR-induced autophagy promotes cell survival or cell death (Paglin et al., 2001; Yao et al., 2003; Ito et al., 2005; Chaachouay et al., 2011; Kim et al., 2011; Wu et al., 2012). Several studies demonstrate that blocking autophagy radiosensitizes, while promoting autophagy radioprotects. The authors argue that IR-induced autophagy is an adaptive response to sustain tumor growth and survival (Chaachouay et al., 2011; Kim et al., 2011). Conversely, other reports show that augmenting IR-induced autophagy increases cell death of radioresistant tumor cells, particularly when an overwhelming amount of autophagy is achieved (Fujiwara et al., 2007; Gewirtz, 2007; Gewirtz et al., 2009; Kuwahara et al., 2011). Undoubtedly, autophagy is a complex response and understanding its role in RT is evolving.

Specifically, the upstream molecular machinery involved in IR-induced autophagy remains unclear (Li et al., 2012). Although IR is known to damage proteins and lipids, IR-induced DNA damage is believed to be the initiating event responsible for autophagy. Recent reports indicate that p53 and PARP-1, a DNA repair enzyme activated by DNA damage, play important roles in autophagy initiation. Both proteins act to inhibit mTOR activity and regulate mTOR's downstream targets, including autophagy (Feng et al., 2005; Huang and Shen, 2009; Rodriguez-Rocha et al., 2011). Interestingly, PARP-1 activation has also been implicated in the necrotic pathway, whereas its caspase-dependent cleavage and inactivation is a downstream event of apoptosis (Huang and Shen, 2009).

**Figure 3:**
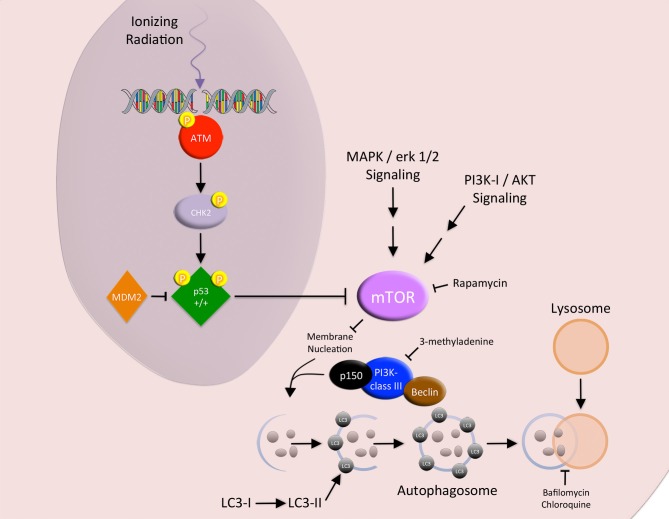
**Autophagy.** The MAPK/erk 1/2 and PI3K-I/AKT signaling pathways block the promotion of autophagy via the activation of MTOR. IR-induced DNA damage activates p53, which acts as a negative regulator of MTOR. Upon initiation of autophagy, the phagophore is generated. A class III PI3K complex (Beclin, class III PI3K, and p150) recruits LC3 to the membrane and facilitates membrane expansion. Complete sequestration by the elongating phagophore results in autophagosome formation. After formation, the autophagosome fuses with the lysosome to become an autophagolysosome, where lysosomal hydrolases digest the sequestered cytoplasmic derived contents.

Upon initiation of autophagy, the phagophore (a nidus for membrane production) is generated either *de novo* or from pre-existing ER membranes (Bernales et al., 2007; Li et al., 2008). A class III PI3K complex (Beclin, Class III PI3K, and p150) recruits LC3 and ATG proteins (ATG12-ATG-5-ATG16L complexes) to the membrane and facilitates membrane expansion. Complete sequestration by the elongating phagophore results in autophagosome formation. After formation, the autophagosome fuses with the lysosome to become an autophagolysosome, where lysosomal hydrolases digest the sequestered cytoplasmic derived contents (Figure 3) (Li et al., 2008, 2012).

Several key proteins regulate autophagy. The canonical class I PI3K/PKB/AKT/mTOR signaling pathway promotes protein synthesis and acts as a negative regulator of autophagy. The binding of insulin/IGF-1 to the insulin receptor has been shown to activate PI3K. Activated PI3K converts Ptdlns(4,5)P2 to yield Ptdlns(3,4,5)P3 at the plasma membrane, leading to PKB/AKT activation. Activated PKB/AKT further activates mTOR (an autophagy inhibitor) through inhibiting the TSC1/TSC2 complex, a repressor of the mTOR activating protein Rheb (Li et al., 2008, 2012; Vellai and Takacs-Vellai, 2010).

Autophagy can be manipulated at several nodes along its pathway. It can be blocked with chloroquine (a lysosomal enzyme inhibitor that reduces autophagosome clearance), Bafilomycin A (a lysosomal proton pump inhibitor that reduces lysosomal acidification and autophagy clearance), 3-MA (a class III PI3K inhibitor), and small interfering RNA to the autophagic machinery (Beclin and the ATG proteins) (Ito et al., 2005; Chen et al., 2011). Conversely, autophagy can be activated with AKT inhibitors and rapamycin, a small molecular inhibitor to mTOR (Fujiwara et al., 2007).

Recent evidence shows that blocking the autophagic machinery with small interfering RNA prevents the release of the immune stimulating “danger signal”, ATP, in chemotherapy treated tumor cells undergoing ICD (Michaud et al., 2011). However, the connection between irradiated tumor cells and their release of ATP as part of an immune stimulating process is currently being defined (Ohshima et al., 2010; Zappasodi et al., 2010).

## Immunogenic cell death

**Figure 4:**
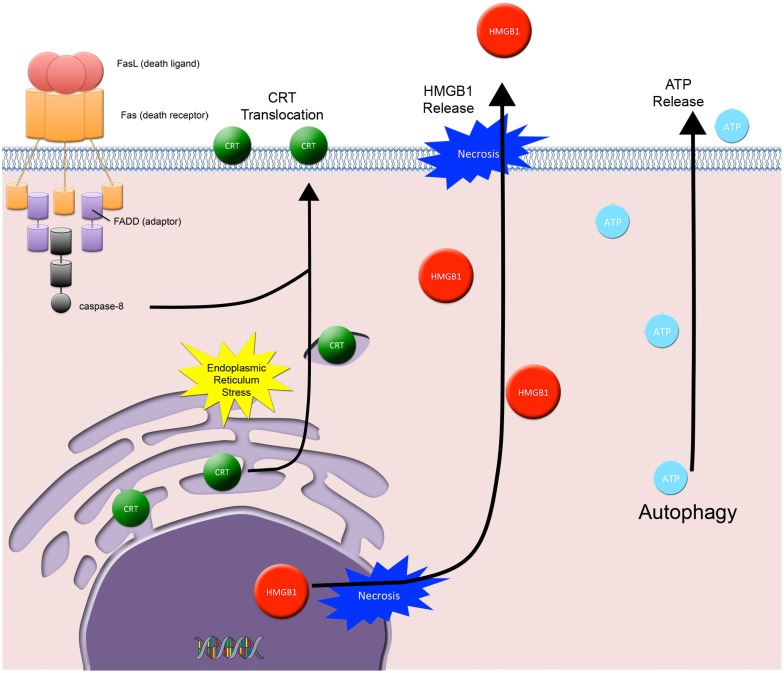
**Immunogenic cell death.** Three distinct arms orchestrate ICD in dying tumor cells and are required for immune priming and activation: **(1)** the cell surface translocation of calreticulin (CRT) and the extracellular release of **(2)** HMGB1 and **(3)** ATP. CRT cell surface exposure acts as a dendritic cell “eat me signal” and involves the coordinated activation of 3 specific modules: ER stress, apoptosis, and ER-Golgi trafficking and extracellular exposure of CRT. HMGB1 is passively released from dying tumor cells and acts as a cytokine and danger associated molecular pattern protein that mediates responses to infection, injury, and inflammation. ATP is released from dying tumor cells and involves the autophagic machinery. Extracellular ATP activates the dendritic cell (DC) P2X7 receptor, which is involved in the upregulation and activation of the DC inflammasome.

Three distinct arms orchestrate ICD in dying tumor cells and are required for immune priming and activation: (1) the cell surface translocation of calreticulin (CRT, an ER residing protein chaperone and potent DC “eat me” signal), and the extracellular release of (2) HMGB1 (a DNA binding protein and TLR-4 mediated DC activator) and (3) ATP (an activator of the DC P2X7 purinergic receptor that triggers DC inflammasome activation, secretion of IL-1β, and subsequent priming of IFNγ producing CD8^+^ T cells) (Figure 4) (Ma et al., 2010). Whereby, the net effects of all three arms act to promote DC phagocytosis of tumor cells, processing of tumor-derived antigens, and DC-associated cross-priming of CD8 + CTLs. However, to date a direct causal link between radiation-induced ICD and an abscopal effect involving the immune system has not been established (Demaria et al., 2004; Dewan et al., 2009). Thus, the challenge remains in understanding the role of radiation-induced ICD and whether or not manipulation of this subroutine of cell death has any significant clinical implications.

CRT cell surface exposure, as described by Kroemer and Zitvogel, is a DC “eat me” signal that involves the coordinated activation of three specific modules: ER stress, apoptosis, and CRT/ERp57 translocation (Panaretakis et al., 2009). The ER stress module requires eIF2 phosphorylation (a marker for ER stress and translation inhibition). The apoptotic module requires caspase-8 activation, Bap31 cleavage, and Bax/Bak activation. Lastly, the translocation module requires anterograde ER-Golgi trafficking and extracellular exposure of CRT/ERp57. Recent studies show that cell surface CRT translocation occurs in IR exposed tumor cells (Obeid et al., 2007; Perez et al., 2009).

In contrast to CRT, cell surface CD47 (a DC “don't eat me” signal) is widely expressed in solid and hematogenous tumor cells (Willingham et al., 2012). CD47 was discovered on newly formed circulating red blood cells (RBCs) and shown to prevent RBC clearance by the splenic reticuloendothelial system (Khandelwal et al., 2007). CD47 blockade of tumor cells and normal tissues is, respectively, associated with immune mediated tumor rejection and radioprotection (Maxhimer et al., 2009; Willingham et al., 2012). However, the role of CD47 in response to IR in tumor cells is yet to be determined.

HMGB1 is an evolutionary conserved nuclear protein that is expressed by almost all cells (cells with an intact nucleus) and is important for the regulation of transcription (Lotze and Tracey, 2005). When released from dying cells, it acts as a cytokine and danger associated molecular pattern (DAMP) protein that mediates responses to infection, injury, and inflammation; thus HMGB1 has been called by Lotze and Tracey the immune system's “nuclear weapon” (Lotze and Tracey, 2005). HMGB1 released from tumor cells binds to TLR4 on DCs, thus contributing to DC activation (Apetoh et al., 2007).

HMGB1 is released into the extracellular space from cells in one of two ways: either actively or passively. Active release involves HMGB1 hyperacetylation in the nucleus followed by vesicular secretion into the immunological synapse or into the extracellular space. HMGB1 is actively secreted by activated macrophages, mature DCs, and activated NK cells (Lotze and Tracey, 2005). In contrast, tumor cells passively release HMGB1 when they undergo either sustained autophagy, late apoptosis, or necrosis (Lotze and Tracey, 2005). Passively released HMGB1 signals through RAGE, TLR2, and TLR4, where it promotes the transcription of pro-inflammatory genes in immune cells.

In addition to promoting an inflammatory response in immune cells, extracellular HMGB1 can trigger autophagy or apoptosis in bystander cancer cells, depending on its redox state. Reduced HMGB1 binds to RAGE, induces Beclin dependent autophagy and promotes resistance to IR and chemotherapy in pancreatic and colon cancer cells (Tang et al., 2010). In contrast, oxidized HMGB1 increases the cytotoxicity of these agents and induces apoptosis via the mitochondrial pathway (Tang et al., 2010). Currently, the redox state of HMGB1 released from IR exposed tumor cells has not been determined.

ATP release is yet another important ICD component. It involves the autophagic machinery, where knock down of ATG7 and ATG5 blocks ATP release (Michaud et al., 2011). Recently, IR has been shown in several models to cause the release of ATP from dying tumor cells and activation of immune cells via the P2X7 purinergic receptor pathway (Ohshima et al., 2010; Zappasodi et al., 2010). This pathway involves the ATP-P2X7 receptor stimulation followed by upregulation and activation of the DC inflammasome (a large multiprotein complex composed of NLRP3, Cardinal, the adaptor ASC, and pro-caspase-1). DC inflammasome activation results in the synthesis and secretion of IL-1β, where secreted IL-1β initiates further pro-inflammatory events (Petrovski et al., 2011).

## Abscopal radiation responses

RT is employed as a local treatment modality with the intent to kill tumor cells and reduce local recurrence. Considerable evidence demonstrates that RT effects extend beyond the treatment field (Formenti and Demaria, 2009). As mentioned earlier, the abscopal effect is a term used to describe tumor regression in lesions outside of the treatment field when one tumor site is irradiated. Known for almost 60 years as a rare unexplained phenomenon in patients receiving local RT (Mole, 1953), it could be the result of RT-induced ICD that generates an *in situ* vaccine (Ma et al., 2010). In support of this notion, interventions that promote the functionality of DCs or improve T cell activation induce the abscopal effect in an unfavorable tumor microenvironment, where the effect is otherwise unseen (Chakravarty et al., 1999; Demaria et al., 2004, 2005). This strongly suggests that, while RT may be efficient at releasing tumor antigens, the immunosuppressive tumor microenvironment may hamper the development of therapeutically effective anti-tumor immune responses.

Additional evidence supports the hypothesis that local RT induces immune-mediated systemic anti-tumor effects. For instance, the well documented association between optimal local control and survival in several breast cancer trials, at a time when occult systemic disease is often already present implies the induction of a systemic anti-tumor mechanism by local RT. In fact, two meta-analyses of prospective randomized trials on the effects of local radiotherapy for breast cancer determined that a 20% absolute reduction in 5-year local recurrence led to a 5% absolute reduction in 15-year breast cancer mortality (a four-to-one ratio of absolute effects) (Clarke et al., 2005; Darby et al., 2011).

Since some chemotherapy drugs can also induce ICD (e.g., Mitoxantrone, Adriamycin, and Oxaliplatin) (Garg et al., 2010; Zitvogel et al., 2010; Kepp et al., 2011; Kroemer et al., 2011), it is intriguing to consider if the superiority of concomitant versus sequential chemo-radiation is due to a synergistic induction of ICD (Glynne-Jones and Hoskin, 2007; Formenti and Demaria, 2008). Chemotherapy-induced ICD was found to be a non-mutually exclusive subroutine of tumor cell death that includes components of the apoptotic, autophagic, and necrotic machineries (Garg et al., 2010; Zitvogel et al., 2010; Kepp et al., 2011; Kroemer et al., 2011). Prior to dying, tumor cells exposed to ICD-inducing drugs were shown to release pro-inflammatory cytokines and alter their display of cell surface antigens, thereby becoming less tolerogenic and more immunogenic (Green et al., 2009). These dying tumor cells were able to prime the immune system of mice and prevent tumor reestablishment when the immunized mice were subsequently re-challenged (Tesniere et al., 2010; Michaud et al., 2011).

Interestingly, patients with breast cancer who are treated with chemotherapy and radiotherapy and carry a TLR4 loss-of-function allele relapse faster than those carrying the normal TLR4 allele (Apetoh et al., 2007). Thus, HMGB1-TLR4 DC signaling is a clinically relevant immunoadjuvant pathway triggered by tumor cell death (Apetoh et al., 2007).

## Future directions

Whether RT specifically and efficiently elicits ICD remains a critical research question. Most of the work of ICD has been described with the use of chemotherapeutic compounds. However, emerging clinical evidence has renewed interest in studying the mechanisms of IR-induced ICD.

Several reports have shown that IR and chemotherapeutic agents induce “danger signals” that may contribute to an immune-mediated response at the tumor site, thereby reverting the immunosuppressive microenvironment of established tumors (Ma et al., 2010). IR and chemotherapeutic agents act to promote an anti-tumor immune response in the tumor microenvironment via ICD pathways, triggering the cross-presentation of tumor-derived antigens by DCs (Ma et al., 2010). However, in the clinical setting each treatment alone may not quantitatively and/or qualitatively achieve tumor cell death in the manner that triggers immune-mediated tumor rejection. Thus, further studies are needed to determine the optimal IR and chemotherapeutic treatments that reposition each other to optimally elicit ICD.

### Conflict of interest statement

The authors declare that the research was conducted in the absence of any commercial or financial relationships that could be construed as a potential conflict of interest.
